# Computed tomographic angular measurements using a bone-centered three-dimensional coordinate system are accurate in a femoral torsional deformity model and precise in clinical canine patients

**DOI:** 10.3389/fvets.2023.1019216

**Published:** 2023-04-17

**Authors:** Andreas Brühschwein, Bronson Schmitz, Martin Zöllner, Sven Reese, Andrea Meyer-Lindenberg

**Affiliations:** ^1^Clinic of Small Animal Surgery and Reproduction, Centre of Veterinary Clinical Medicine, Veterinary Faculty, LMU Munich, Munich, Germany; ^2^Department of Veterinary Sciences, Veterinary Faculty, Institute of Veterinary Anatomy, Histology, and Embryology, LMU Munich, Munich, Germany

**Keywords:** dog, femur, computed tomography, 3D coordinate system, torsion, torsional deformity model

## Abstract

**Introduction:**

In small animal orthopedics, angular measurements in the canine femur are often applied in clinical patients with bone deformities and especially in complex and severe cases. Computed tomography (CT) has been shown to be more precise and accurate than two-dimensional radiography, and several methods are described. Measurement techniques evaluated in normal bones must prove accuracy in deformed bones in clinical settings.

**Objectives:**

The goals of our study were to evaluate the accuracy of canine femoral torsion angle measurements in a femoral torsional deformity model and to test repeatability and reproducibility of canine femoral neck inclination, torsion, and varus angle measurements in CT datasets of dogs applying a CT-based technique using a three-dimensional (3D) bone-centered coordinate system.

**Materials and methods:**

For precision testing, femoral torsion, femoral neck inclination, and femoral varus angles were measured in CT data of 68 canine hind limbs by two operators, and their results were compared. For accuracy testing, a femoral torsional deformity model was preset from 0° to +/−90° with a goniometer and scanned. Torsion angles were measured in the CT data and compared to the preset value.

**Results:**

In the femoral torsion model, the Bland–Altman plots demonstrated a mean difference of 2.11°, and the Passing–Bablok analysis demonstrated a correlation between goniometer and CT-based measurements. In the clinical CT scans, intra- and interobserver agreement resulted in coefficients of variation for repeated measurements (%) between 1.99 and 8.26 for the femoral torsion, between 0.59 and 4.47 for the femoral neck inclination, and between 1.06 and 5.15 for the femoral varus angles.

**Discussion:**

Evaluation of femoral malformations with torsional deformities is the target area of this technique. Further studies are required to assess its value in different types, degrees, and combinations of osseous deformities and to establish normal reference values and guidelines for corrective osteotomies.

**Conclusion:**

Based on the results of this study, the accuracy of the torsion angle measurements and the precision of inclination, torsion, and the varus angle measurements were considered acceptable for clinical application.

## 1. Introduction

In small animal orthopedics, angular measurements in the canine femur are often applied in dogs with patellar luxation (1–16) or severe posttraumatic bone deformity (17–20), and are commonly performed using radiography (9, 13, 14, 20, 21). Radiographs are two-dimensional images that lack a third dimension and are flawed by geometric errors based on projection, summation, magnification, and distortion (22). Standardized radiographic positioning is used to minimize geometric and angular measurement errors. Minor projectional deviations cause variation in angular measurements (18, 23–27). This might affect the results unpredictably, as shown for canine distal femoral varus angle measurements (26). The term *varus* is used for the determination of lateromedial deviation of the bone axis within the dorsal (frontal) plane. The *transverse plane* is perpendicular to the long axis of the body or body parts (28, 29). The *sagittal plane* is parallel to the median plane (28, 29). The *dorsal plane* is parallel to the dorsal surface of the body or body parts and is perpendicular to the median (sagittal) and transverse planes (28, 29). In limbs, the synonymous term “*frontal”* is commonly used but not recommended by the current Nomina Anatomica Veterinaria (29). In the transverse plane, a diaphyseal twist of the shaft is commonly referred to as femoral torsion. The term femoral (neck) version is used to describe the orientation of the femoral neck in relation to the femoral condyles (30). In man, femoral torsion and femoral version can be distinguished (31). No reliable features are described to differentiate both entities in dogs, and these terms are often considered synonymous (31–33). In the normal femur, the femoral neck and head are oriented craniomedially termed antetorsion or anteversion. An abnormal caudal deviation is called retrotorsion or retroversion. Radiographic distoproximal projections of the femur to determine femoral torsion angles can be difficult to obtain, and additional fluoroscopic guidance should be used (30). Alternatively, biplanar radiographic methods with mathematical correction or calibration curves are described (32, 34–37).

Computed tomography (CT) generates true three-dimensional (3D) information and is used to overcome the limitations of two-dimensional radiographic projection errors (2, 8, 10–12, 16, 18, 19, 27, 33, 35, 37–47). Similar to radiography, standardized positioning is a prerequisite for several CT techniques (7, 8, 38, 39). If the reference points for angular measurements are located in different CT images, postprocessing of the cross-sectional images is required. Summation of two individual CT images into one single superimposed image (40, 43, 48, 49), multiplanar reformations (8, 33, 35, 39), maximum intensity projections (MIP) (39), or volume rendering (VR) techniques (12, 17–19, 27, 31, 33, 37–39, 41–43) are applied to enable measurements within a two-dimensional image. MPR, MIP, or VR allow free rotation and choice of perspective, but based on the final selected view, a planar image is created that lacks a third dimension. Points and lines that define the angle are coplanar in the postprocessed and reconstructed CT image but still remain as skew lines in the three-dimensional anatomy of the patient (50, 51). Based on the potential variation of the selected measurement perspective, projectional variation and error remain. This is limiting angular measurements in complex angular and torsional deformities (18).

Angular measurement techniques heavily rely on standardized positioning, projection, or perspective that can be easily achieved in normal bones or normal cadavers but can be difficult in animals that suffer from an orthopedic disease with deformed bones or restricted articular ranges of motion. Many radiographic and computed tomographic techniques were established in normal individual bones or normal cadavers only (2, 25–27, 30, 32–34, 39, 40, 42, 44, 52–58). Accuracy and precision of measurements in normal cadaver bones do not necessarily prove the validity of a measurement technique in deformed bones and in patients in a clinical setting. Therefore, the goals of this study were to evaluate the accuracy of a three-dimensional CT-based technique using a 3D coordinate system to measure canine femoral torsion angles in a canine femoral torsional deformity model and to test precision using intraobserver variability (repeatability) and interobserver variability (reproducibility) of femoral torsion, femoral neck inclination, and femoral varus angles in CT data of dogs that were scanned in a clinical setting.

## 2. Materials and methods

### 2.1. Femoral torsional deformity model

**Figure 1:**
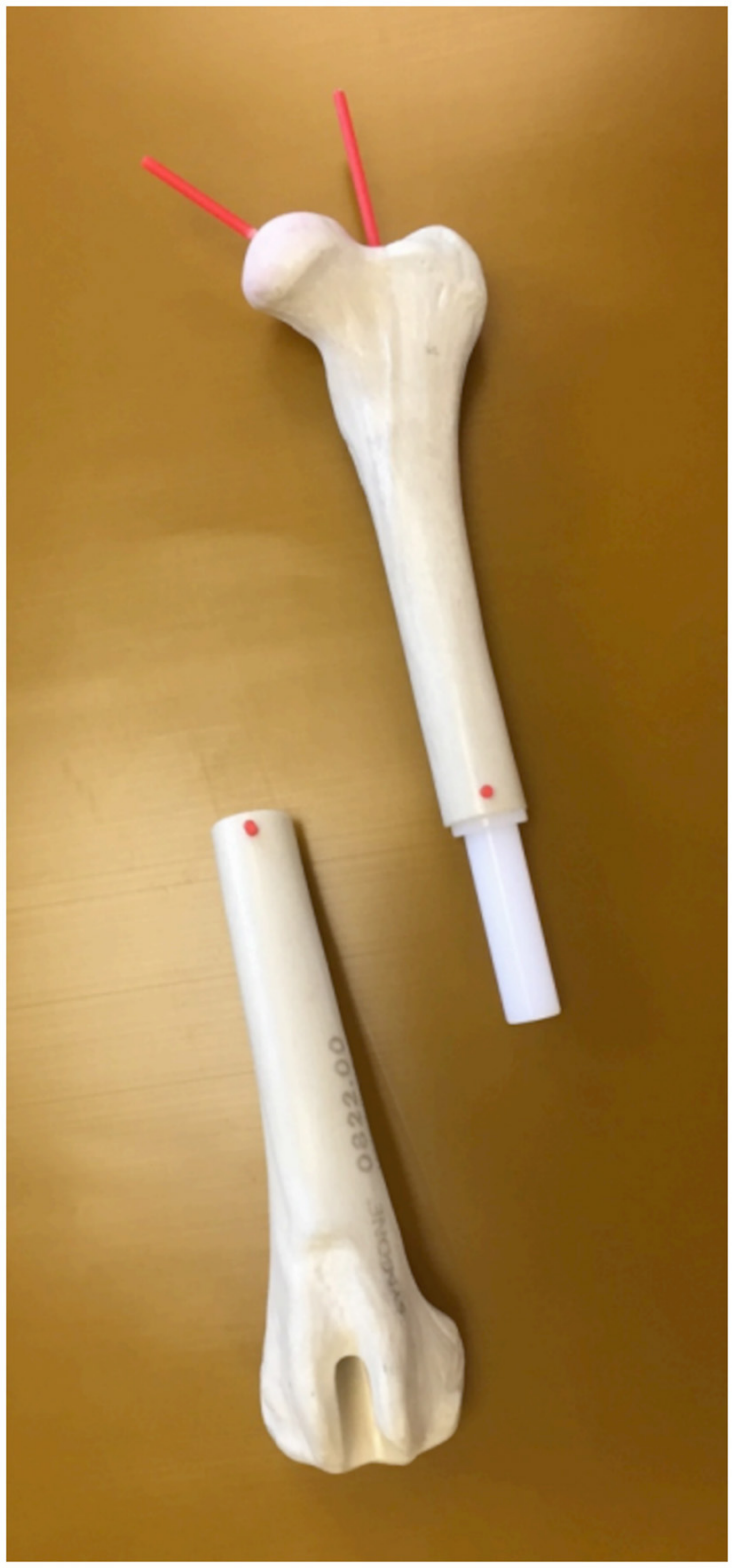
Torsional deformity model. An anatomical femoral bone model of a normal canine femur (Synbone^®^, Malans, Switzerland) with a mid-diaphyseal rotational hinge enabled free twisting of both segments about the femoral longitudinal axis.

**Figure 2:**
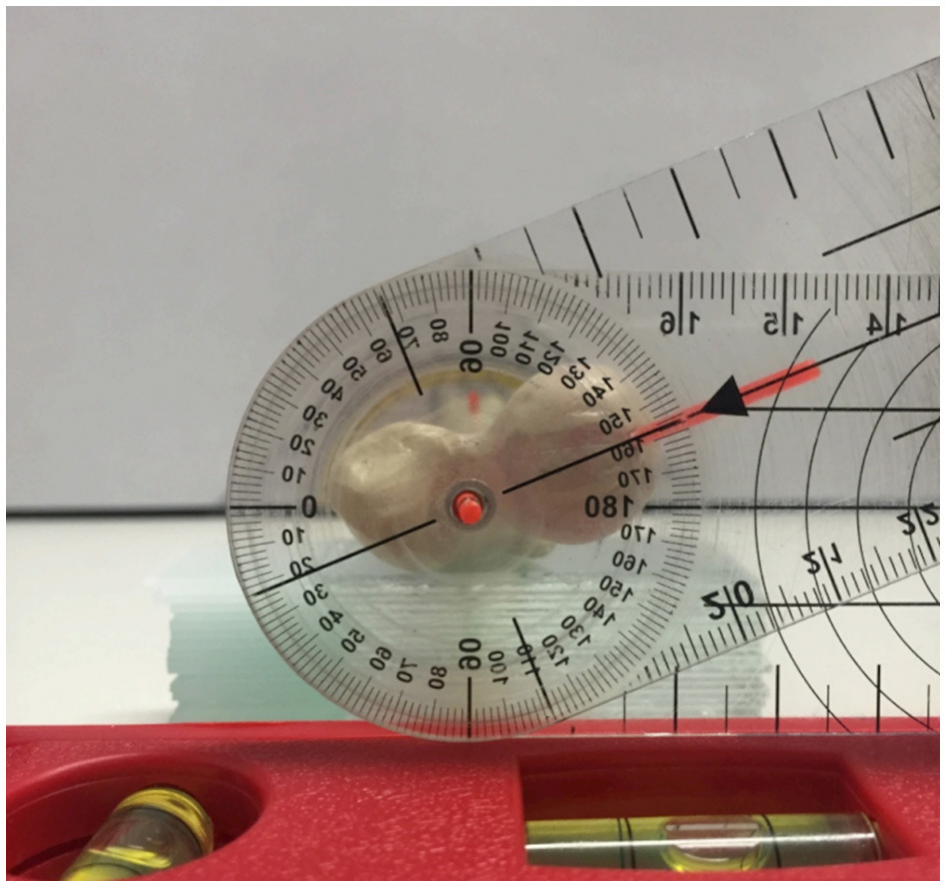
Manual goniometer measurements of the femoral torsion angle. Proximodistal view on the torsional deformity model with the pivot point of the goniometer inserted into the femoral longitudinal axis rod. The legs of the goniometer are aligned with the retrocondylar axis (spirit level) and the plastic rod of the femoral neck axis.

In the first part of the study, a femoral torsional deformity model was constructed to serve as a reference standard for the determination of the accuracy of the software measurements. An anatomical bone model of a normal canine femur (Synbone^®^, Malans, Switzerland) was cut in a transverse plane at the mid-diaphyseal level. A plastic rod suitable in diameter was introduced into the round medullary cavity and served as a hinge for rotation around the femoral longitudinal axis. Two drilled-in cranial cortical markers defined the initial normal physiological baseline position. Holes were drilled, and thin plastic rods were inserted in a direction that matched the long axis of the femoral neck and the diaphyseal axis at the proximal aspect of the model (Figure 1). Goniometer measurements were intended to serve as a reference standard for comparison with software measurements in CT data, so the precision of goniometer measurements was evaluated first. A total of 10 torsion angles of the femur were randomly set by blindly twisting the model by one person. These preset torsion angles were measured independently by two observers (BS and AB), with the other observer's result unknown. Femoral torsion angles were measured using a transparent plastic goniometer with a 1° reading increment (Rulongmeter style). The femoral diaphysis was placed horizontally with the condyles in contact with the table using a spirit level. The pivot point of the goniometer was inserted into the rod of the longitudinal femoral axis. One leg of the goniometer was supported with a spirit level parallel to the retrocondylar axis. The second leg was aligned parallel to the plastic rod of the femoral neck axis (Figure 2). Interobserver variability (reproducibility) was analyzed by calculating the *coefficient of variation for repeated measurements* with the goniometer. In the next step, the femoral torsional model was rotated and preset for comparison with the software measurements. The physiological femoral neck antetorsion angle of +21.5° corresponded to the normal 0° setting of the diaphyseal rotary joint of the torsional deformity model. Starting from this 0° position, the rotary joint was set at +/−10°, +/−20°, +/−30, +/−40°, +/−50°, and +/−90°. Goniometer measurements of the rotary joint and the femoral neck torsion angle with the consensus agreement of two operators were used to define the reference standard in the bone phantom. CT scans were performed in each simulated degree of torsional deformity for comparison with the software measurements.

### 2.2. CT scanning parameter and software

For all CT examinations in this study, a helical multi-slice scanner with a fixed detector array design (SOMATOM Definition AS VA48A_02_P12, 64 Excel Ed. software Somaris/7 syngo CT VA48A Siemens Healthcare GmbH, Erlangen, Germany) was used. Scans were performed in helical mode. Scanning slice thickness was 0.6 mm, tube voltage was 120 kV, tube rotation time was 0.5–1s, pitch was 0.8, and tube current was variably adjusted according to the size of the object or patient. The reconstructed slice thickness and increment were 0.6 mm. Images were reconstructed using a bone algorithm (deconvolution filter: kernel 60 or 70). The Digital Imaging and Communications in Medicine (DICOM) data of the CT scanner were exported to a network-attached storage for further use by medical imaging software and anonymized regarding the preset torsion angles. Medical imaging software VoXim^®^ (version 6.5.1.1 (T2160910) Copyright) from the company IVS Technology GmbH (LLC), Chemnitz, Germany, was used for the 3D angular measurements. DICOM image data were imported into the program. The software was designed for 3D image-guided surgery, had medical device approval, and was used in prior research. MPR, VR, bone segmentation, and a 3D coordinate system were the main features used for angular measurements as previously described for normal canine femurs (58).

### 2.3. Accuracy of 3D torsion angle measurements in the torsional deformity model

Torsion angle measurements in the CT scans of the variably twisted torsional deformity model were performed and compared with the preset angle. When setting the measurement points in the CT images, the result of the measurement could not be seen. Femoral torsion angles were calculated using a three-dimensional method with a bone-centered 3D coordinate system and projection of the femoral neck and retrocondylar axis into a plane transverse to the femoral longitudinal axis as described earlier (58). The femoral neck axis was defined by two reference points. The femoral head center was the midpoint of a 3D semiautomatic femoral head fitting sphere along its bearing surface. The femoral neck base center was set directly using a circular crossline tool at the midpoint of the femoral metaphysis at the level of the lesser trochanter (58). The retrocondylar axis was determined by the lateral and the medial femoral condyle center at the most caudal aspect of the subchondral bone surface (58). The software measured the torsion angles in the transverse plane using 3D coordinates of the anatomical reference points.

### 2.4. Precision of 3D angle measurements in canine patients in a clinical setting

In the second part of the study, intraobserver variability (repeatability) and interobserver variability (reproducibility) of femoral angular measurements based on a 3D coordinate system and vector geometry were tested in CT scans of dogs. We queried the digital diagnostic imaging archive (dicomPACS^®^, Oehm und Rehbein, Rostock, Germany) of the clinic. We searched and retrieved studies of client-owned canine patients with a clinical diagnosis of patellar luxation that underwent CT examinations of both hind limbs for routine preoperative assessment and planning of surgical correction with the consent of the owner between 2012 and 2015. These dogs were scanned prior to surgery under general anesthesia in dorsal recumbency with extended hindlimbs using foam pads, velcro strips, and tapes. The position was similar to a ventrodorsal pelvic radiograph for canine hip dysplasia screening, having extended coxofemoral, stifle, and tarsal joints with the hind limbs' longitudinal axes parallel to the lumbar spine. 3D angular measurements were performed in the CT data by two observers (AB and BS) using the VoXim^®^ 3D imaging software. One operator (BS) repeated all measurements after 6 weeks. All measurements were performed independently and in a blinded fashion. Femoral torsion (FTA), femoral (neck) inclination (FIA), and femoral varus angles (FVA) were measured as described earlier (58). Two alternative variations of the femoral torsion and neck inclination angles were measured using two different femoral neck axes as follows: first, between the femoral head center (FHC) and the femoral neck center (FNC), and second, between the femoral head center (FHC) and the femoral neck base center (FNBC) (58). Femoral varus (or valgus) angles (FVA) were also measured in two variations, using the distal femoral longitudinal axis in combination with a proximal (pd) and alternatively with a total (td) femoral longitudinal axis (58).

### 2.5. Statistical analysis

Torsion angles measured with the 3D imaging software VoXim^®^ in the CT data were compared with the preset angles in the torsional deformity model using the Passing–Bablok regression analysis (59–61) and the Bland–Altman plots (62, 63). To estimate the repeatability (intra- and interobserver variability) of angular measurements, the *coefficient of variation (CV) for repeated measurements* was calculated according to Bland (63). To estimate the intraobserver variability, the *CV for repeated measurements* of the same observer was calculated. To estimate the interobserver variability, the *CV for repeated measurements* was calculated for the same measurements of different observers. *CVs for repeated measurements* were considered excellent < 3%, good < 10%, moderate/fair < 15%, and poor >15%. The statistical analysis was performed using the software IBM SPSS 23 and MedCalc 20.111.

## 3. Results

### 3.1. Femoral torsional deformity model

In the femoral torsion model, the 10 randomly and blindly set torsion angles ranged between 2° and 84°. Before the use of CT, the comparison between two goniometer measurements (Figure 2) by two observers (interobserver agreement) of the same twisted femoral torsion model revealed a coefficient of variation for repeated measurements of 2.9%. The individual results of the goniometer measurements of the two observers (BS and AB) are shown in Supplementary Table S1.

**Figure 3:**
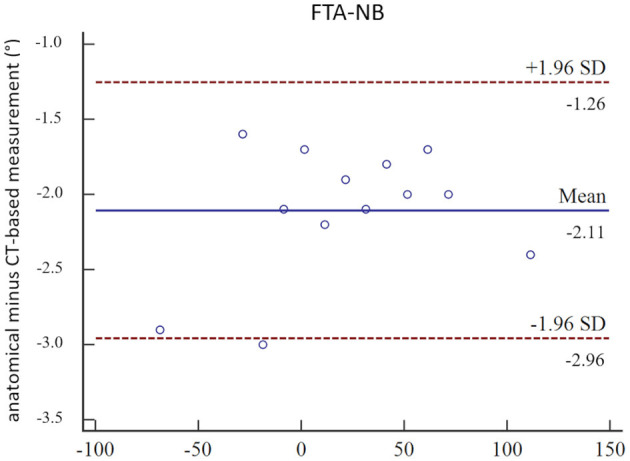
Bland–Altman plots. Bland–Altman plots of the comparison between manual anatomical (goniometer) and CT-based (software) femoral neck version angle measurements (FTA-NB) in a canine femoral diaphyseal torsional deformity model in 13 preset femoral torsion angles.

**Figure 4:**
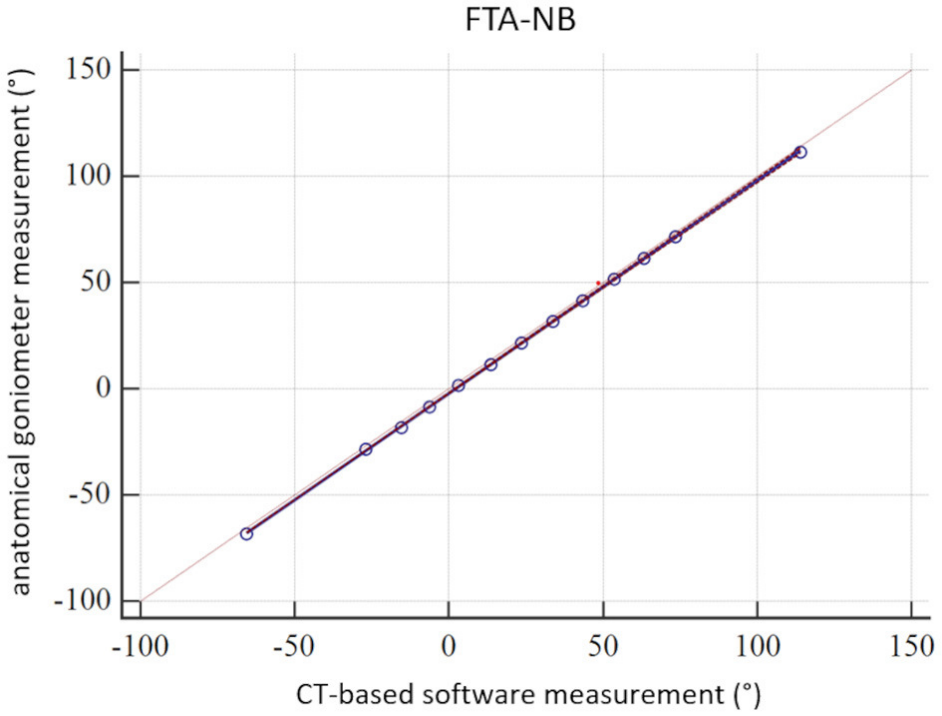
Passing–Bablok analysis. Scatter plot and regression line of the Passing–Bablok analysis of the comparison between manual anatomical (goniometer) and CT-based (software) femoral neck version (torsion) angle measurements (FTA-NB) in a canine femoral diaphyseal torsional deformity model in 13 preset femoral torsion angles.

In the CT scans of the femoral torsional deformity model, the anatomical reference points could be set by the operator, and the software could calculate the angles. The results of the comparison between the goniometer and CT-based software measurements in the torsional deformity model were between −68.5° and 113.9° and are shown in Table 1. The goniometer measurements were systematically slightly lower than the CT-based digital software measurements (Table 1). The Bland–Altman plots of the comparison between goniometer and CT-based measurements revealed a 2.11° mean difference with the software measurement being higher than the goniometer measurements (Figure 3). Scatter plots and regression line of the Passing–Bablok analysis of the comparison between goniometer and CT-based measurements are shown in Figure 4. The 45° straight linear slope of the Passing–Bablok regression analysis demonstrates the correlation between both methods (Figure 4).

**Table 1:** Results of the femoral neck version angle measurements in the femoral torsion model.

**Comparison between goniometer and CT-scan based software measurements of the femoral neck version angle in a canine femoral torsional deformity model in physiological (0** **°** **/21.5** **°** **) and abnormal preset diaphyseal torsion angles**		
**Goniometer**		**Software**
**Preset measurement rotation hinge angle**	**Preset measurement femoral torsion angle**	**CT-scan measurement femoral torsion angle (NBC)**
−90°	−68.5	−65.6
−50°	−28.5	−26.9
−40°	−18.5	−15.5
−30°	−8.5	−6.4
−20°	1.5	3.2
−10°	11.5	13.7
0°	21.5	23.4
+ 10°	31.5	33.6
+ 20°	41.5	43.3
+ 30°	51.5	53.5
+ 40°	61.5	63.2
+ 50°	71.5	73.5
+ 90°	111.5	113.9

### 3.2. 3D angle measurements in canine patients in a clinical setting

For the evaluation of the measurement technique on clinical data from the picture archiving and communication system of the clinic, we found bilateral pelvic limb CT studies with extended hind limbs of 34 clients that matched the technical requirements of thin-sliced gapless three-dimensional data volumes reconstructed with bone algorithms. We used 34 right (r) and 34 left (l) pelvic limbs in 34 CT datasets for the 3D angular measurements.

For the femoral torsion (FTA) and femoral (neck) inclination (FIA) angles, coefficients of variation for repeated measurements (%) were below 5.2 for the intraobserver agreement and below 5.6 for the interobserver agreement, with exception of the FTA-NC that was 8.3 for the left side and 8.2 for the right side. Coefficients of variation for repeated measurements (%) of the torsion angles were lower for the FTA-NBC (2.0/2.6 and 5.4/5.8) than for the FTA-NC (3.4/3.0 and 8.3/8.2) for intra- and interobserver agreement, respectively. Femoral neck inclination angles had lower coefficients of variation for repeated measurements (%) than torsion angles. Angles based on the reference point *neck base center* (NBC) had lower coefficients of variation for repeated measurements than those based on the reference point *neck center* (NC), with the exception of one outlier (left-sided FIA-NBC intraobserver agreement). For the femoral varus (or valgus) angles (FVA), intra- and interobserver agreement revealed coefficients of variation for repeated measurements (%) between 1.06 and 2.10, except for the right-sided FVA-td (r) intraobserver agreement of 5.146.

FTA-NBC was higher than FTA-NC. Differences in their means were 2.16° and 2.25° for the left-sided and 3.51° and 3.47° for the right-sided repeated intra- and interobserver measurements. FIA-NBC was higher than FIA-NC. Differences in their means were 19.86° and 19.54° for the left-sided and 19.20° and 19.01° for the right-sided repeated intra- and interobserver measurements, respectively. FVA-td angles were smaller than FVA-pd. Differences in the means were 2.17° and 1.36° for the left-sided and 1.53° and 0.33° for the right-sided repeated intra- and interobserver measurements, respectively. All results of the intra- and interobserver agreement calculations are shown in Table 2. The individual measurement results of each dog and both observers (O1 and O2) as well as observer one (O1) at two occasions [O1(1) and O1(2)] are shown in Supplementary Table S2.

**Table 2:** Intra- and interobserver agreement of CT-based femoral angular measurements in a clinical setting in 34 dogs with a clinical diagnosis of patellar luxation that underwent preoperative CT examinations of both hind limbs for routine preoperative assessment and planning of surgical correction.

**Angle**	**Intraobserver agreement**			**Interobserver agreement**		
**(l)** = **left hindlimb** **(r)** = **right hindlimb**	**Mean degree (**°**)**	**Standard deviation**	**Coefficient of variation for repeated measurements (%)**	**Mean** **degree (**°**)**	**Standard deviation**	**Coefficient of variation for repeated measurements (%)**
FTA-NBC (l)	22.196	0.442	1.993	22.175	1.202	5.418
FTA-NBC (r)	23.766	0.623	2.622	23.818	1.374	5.77
FTA-NC (l)	20.038	0.688	3.431	19.922	1.646	8.264
FTA-NC (r)	20.256	0.606	2.992	20.349	1.66	8.158
FIA-NBC (l)	137.84	6.1646	4.472	137.077	1.595	1.163
FIA-NBC (r)	137.249	0.62	0.452	137.104	1.429	1.042
FIA-NC (l)	117.918	1.119	0.949	117.593	2.079	1.768
FIA-NC (r)	118.052	0.696	0.59	118.096	1.695	1.435
FVA-td (l)	173.835	3.306	1.902	173.768	2.771	1.595
FVA-td (r)	173.579	8.993	5.146	174.606	2.863	1.64
FVA-pd (l)	176.007	3.694	2.099	175.129	1.859	1.061
FVA-pd (r)	175.104	3.384	1.933	174.937	2.931	1.676

Femoral torsion (FTA) and femoral (neck) inclination (FIA) angles were measured in two variations, using the femoral head center in combination with the femoral neck base center (FNBC), and with the femoral neck center (FNC). Femoral varus (or valgus) angles (FVA) were measured in two variations, using the total (t) with the distal (d), and the proximal (p) with the distal (d) femoral longitudinal axis. Left (l) and right (r) pelvic limbs.

## 4. Discussion

The main results from this study demonstrate the accuracy of CT-based torsion angle measurements in a bone phantom over a range of 180° femoral torsional deformity, and the precision of femoral (neck) inclination, torsion, and varus angle measurements in CT scans of clinical canine patients. A bone-centered coordinate system in alignment with the anatomical transversal and dorsal planes of the bone defined the projection planes for true 3D angle measurements (58). Instead of visually guided virtual positioning of a 3D CT bone image, 3D coordinates and mathematical definitions were used to standardize the measurement. The advantage of a true 3D technique should be most apparent in bones that are severely deformed in multiple planes. However, we have not yet proven this hypothesis with our single transverse plane bone deformity model. Studies on normal bones might not reveal these possible advantages. The use of a 2D technique might be sufficient, if a bone is straight and normal, or is deformed in one single plane only.

### 4.1. Femoral torsional deformity model

The use of a plastic bone model instead of a normal bone for the construction of the torsion model could be a possible limitation of these experiments. However, the plastic bones and their surfaces were clearly visible on the CT scans. Therefore, we believe that the use of the phantom did not result in significant error or bias. The reason for using the model was the availability and the fact that we could experiment, prototype, and work with the plastic bone models very easily without needing or wasting bones from animal cadavers. The creation of the torsional deformity model required a few repeated prototyping processes for the rotational hinge and the position and direction of the drill holes for the plastic rods to attach the goniometer and measure the femoral neck axis. The drill hole for the plastic rod of the femoral neck was tried to align as well as possible along the axis of the femoral neck to create the reference standard and to determine the angles in a similar way to other authors (6, 7, 30, 33). Mild malalignment of the plastic rods in the torsional deformity model might explain the systematic differences of 2.11° between the goniometer and the software measurements (Table 1, Figure 3). The alignment of the femoral neck rod and the goniometer measurements in the torsional deformation model were more similar to the CT-based FTA-NC angle measurements than to FTA-NBC. In the clinical patients (Table 2, Supplementary Table S2), a similar mild systematic difference between FTA-NC and FTA-NBC was found. We, therefore, assume that these minor differences in femoral neck reference points and axes are the main reasons for the systematic differences of 2.11° between the goniometer and the software measurements.

Perfect accuracy and precision would be ideal and should be aimed, but what is an acceptable size of error? To the best of our knowledge, minimum requirements for an orthopedic angle measurement technique with regard to accuracy and precision are not known to us. A quantitative assessment of the inaccuracy of surgical angle manipulation in a human cadaver study revealed a mean error of 8.8° for Kirschner wire placement using a manual goniometer technique, which could be reduced to 2.1° mean error when a mounted digital goniometer device was used (64). Based on this surgical precision and error size, and as long as no better method is available, we considered the inaccuracy and imprecision of our 3D method acceptable for current clinical application.

Reference values for torsion angles in humans significantly vary based on the CT measurement technique used (65, 66). Therefore, any CT-based femoral torsion angle measurement and reference value should only be used in close connection and with the exact specification of the respective measurement technique and should not be transferred between different techniques (65, 66).

A true gold standard is difficult to determine. We used the goniometer measurements as a reference standard for comparison with CT-based software measurements to assess accuracy in the torsional deformity model. Manual goniometer measurements are not necessarily more accurate or precise than CT-based measurements. Therefore, to get an initial impression of the technique, which should serve as a reference standard, we evaluated the interobserver agreement and thus the precision of the goniometer measurements. In the torsional deformity model, we simulated osseous deformation up to a range of 180° and only in the transverse plane. Bone malalignment is not limited to torsional deformity. It might have additional bowing along the dorsal (varus or valgus) and sagittal (procurvatum or recurvatum) plane. Translational components and other bone malformations that do occur could influence measurement results. Based on our experiment, we do not know whether this technique would be accurate for very severe (>180°) and complex bone deformities.

In canine medicine, reference values for femoral alignment could vary with dog conformation or might even be breed specific (67). In the human femur, the results of torsional deformity measurements did not only vary based on the CT measurement technique that was used but also does seem to depend on the degree of torsional deformity (66). Therefore, measurement techniques that were established and validated for normal bones only might not necessarily provide reliable results in abnormal bones. The validity of measurements might depend on the type and degree of the present bone deformity. To ensure that our CT-based 3D angular measurement method works not only in normal but also in femurs with torsional deformities, one goal of this study was to evaluate the application and accuracy in a torsional deformity model. By twisting the femoral diaphysis of a plastic bone model with a rotation hinge, we simulated normal and abnormal torsion angles and measured the angles based on reference points that are located at the femoral head and neck proximally, and the femoral condyles distally. The femoral neck version denotes the relative orientation of the femoral neck in relation to the femoral condyles (30). We did not use diaphyseal landmarks, and our technique would not distinguish between the femoral neck version and torsion. Currently, we also would consider these two measurements synonymous. Both terms could be used interchangeably (31–33) until we would find valid reference points to distinguish both anatomical entities in the canine patient. To test the accuracy of the measurement technique in torsional deformities, we used a high range (+/−90°) covering 180° of rotation, starting with a normal physiological antetorsion. Due to a lack of a true gold standard, we used a visual goniometer reference standard by consensus agreement of two operators, not aiming for perfect precision or accuracy.

### 4.2. CT scanning parameter and software

We used CT scans with very thin images of 0.6 mm without interslice gaps that were reconstructed with high-frequency bone algorithms. Currently, we do not know how slice thicknesses, interslice slice gaps, or reconstruction algorithms might affect the measurement results. Therefore, care should be taken, especially when using thicker CT images or series with interslice gaps. Precision and accuracy may be different. We have shown that the technique is independent of the positioning of the bones in the scanner gantry (58). Therefore, patient and limb positioning should not play a role. Our clinical patients were positioned with extended pelvic limbs on the scanner table similar to an extended ventrodorsal pelvic hip dysplasia screening radiograph (OFA view). Flexing the limbs might affect the general image quality. Whether and to what extent poor image quality might affect the measurement results is not known.

The necessity of exporting and importing images into extra additional software is a disadvantage of this technique. DICOM is the technical standard commonly used in veterinary diagnostic imaging. Therefore, we consider the use of 3D medical imaging software with official medical device approval as an advantage of this project. Cost and limited accessibility of commercial software are a constraint until free open source software variants are available for this technology. Approval by a medical device law is not currently required for the use of software in animals in many countries, as far as the authors know. This means that veterinarians in many countries are not limited to programs with medical device approval, nor are they limited to DICOM conformant software. The use of other technical standards, such as computer-aided design (CAD), is therefore also possible (42, 44).

Technical differences in the tools used could cause systematic bias and might have an influence on precision expressed by repeatability and reproducibility. Our software included a fixed orthogonal MPR tool, but a freely adjustable double oblique MPR tool or even a curved MPR tool might help and improve to determine reference points in future more easily and consistently. Parallel measurements of identical angles based on identical anatomical reference points, but using different viewer tools to identify and set the reference points, might help to determine superior strategies and to improve precision and accuracy in future. Additional digital tools such as semiautomatic fitting spheres or machine learning will likely improve the current measurement techniques but might also lead to the use of new reference points.

### 4.3. 3D angle measurements in canine patients

In general, torsion angles showed the lowest precision, followed by the varus and inclination angles (Table 2). The size of the absolute measured values has an influence on the differences in percent of the coefficient of variations for repeated measurements since the torsion angles are smaller than the inclination and varus angles. The type of angle calculation could also play a role. Torsion angles required the projection of skew lines into a transverse projection plane and varus angles into a dorsal plane. Geometric projection during the mathematical calculations likely contributes to the precision of the angular measurement. Minor variation when setting the reference point could lead to an amplification of the variation during projection and thus higher measurement error. In contrast, inclination angles were calculated directly between two intersecting axes.

The torsion angles determined with the FNBC were more precise than with the FNC. Technical differences between the tools used to determine the reference points could play a role. The axis between the femoral head center and the femoral neck center was a plumb line to a virtual femoral neck resection plane, and the femoral neck center was set directly. It is difficult to set reference points using transverse CT images only, especially for the femoral neck center and oblique sections. This is probably the cause, why early CT-based techniques used the femoral neck base center as an alternative to the femoral neck center (6, 7). Development of postprocessing techniques enabled new perspectives, views, and measurements using MPR (8, 33, 35, 39), MIP (39), and VR (12, 17–19, 27, 31, 33, 37–39, 41–43). Without free MPR and VR techniques, it is difficult to determine reference points in clinical patients having double oblique osseous cross section due to severe limb deformities or oblique positioning. In future, better tools to localize the femoral neck reference points can achieve improvements in precision. The distance from the FHC to the FNC is shorter than to the FNBC. Based on geometry, the same spatial variation of two reference points causes higher variation in angular measurements, when both reference points are located closer together, than when they are located farther apart. From this, it can be generally assumed that suitable reference points should not be too close to each other. Instead, they should be as far apart as possible. We assume that anatomical quality of reference points, spatial proximity of reference points, types of localization tools, and procedures of geometrical calculation contribute to the precision of angular measurements and potential error. Their proportional quantification is difficult and requires further studies. For the inclination angles, the angle measurement precision with the FNBC was slightly better than that with the FNC, probably due to similar reasons as for the torsion angles. Femoral (neck) inclination and varus angles showed higher precision than torsion angles, probably mostly due to higher absolute angle size, and due to less geometrical projection during the calculation process. Quality of anatomical reference points and larger distances between them might also play a role. Two outliers in the intraobserver agreement are difficult to explain. FIA-NBC (l) was 4.47%, while all other inclination angles resulted in an intraobserver agreement below 1%. Coefficient of variation for repeated measurements was 5.15% for the FVA-td (r), while all other varus angles had a coefficient of variation for repeated measurements below 2.1%. These outliers of the intraobserver agreement were unilateral, and repetitive studies with other observers and other CT data might assist to find the cause. For the FIA-NBC (l), it was probably not caused by variation of the FNBC because the FTA-NBC (l) is low (1.99%). Based on the predefined assessment scale for the coefficient of variation for repeated measurements, the precision of the 3D angular measurements was rated as good.

For the absolute values of the angles, we found differences between FTA-NC and FTA-NBC, as well as between FIA-NC and FIA-NBC, probably based on their different reference points. FTA-NC and FIA-NC do more closely resemble the initial radiographic techniques. The femoral neck inclination angle measured on a radiograph is only accurate and corresponds to the true anatomical cervico-diaphyseal angle, if the femoral neck is positioned parallel to the detector (54). Precise positioning, beam centering, and projection are possible in a single anatomically isolated cadaver bone but are difficult in dogs in a clinical setting. Normally, in a craniocaudal view of the femur of a dog obtained in a clinical setting, the projected angle of inclination on the radiograph is not identical to the true anatomical angle of inclination (52, 68–70) and depends on view, projection plane, and the angle of version (23, 34, 52, 69). Even in computed tomographic VR images, femoral neck angles depend on and vary with the selected view (42).

In our setting, the differences between FIA-NC and FIA-NBC were ~20°, whereas the differences between FTA-NC and FTA-NBC were much smaller with the FTA-NC which was ~2° smaller than FTA-NBC. For the calculation of the torsion angles, the femoral neck axis is projected into the transverse plane, which eliminates the angulation component along the direction of the longitudinal axis of the femur. This explains the higher difference between FIA-NC and FIA-NBC than the difference between FTA-NC and FTA-NBC. FTA-NBC might be used as an alternative to FTA-NC based on the small difference (2°). We do not think that FIA-NBC could serve as an anatomically accurate alternative for FIA-NC, and there was no clinical reason for the calculation of the FIA-NBC. We calculated FIA-NBC as an additional angle to be able to compare its precision.

In the CT data of the dogs that were scanned in a clinical setting, we could not evaluate the accuracy of our measurements. This is a limitation. Instead, we evaluated intraobserver variability (repeatability) and interobserver variability (reproducibility) of the femoral torsion (neck) inclination, and varus angles. For the evaluation, we used clinical CT data to ensure that the technique is robust in clinical scans. These included cases with bone deformities and therefore variable positioning of extended hind limbs. For the femoral neck inclination and distal femoral varus angle measurements, we did not evaluate accuracy by direct comparison between CT and goniometer measurements. Even if each of these angular measurements would prove to be accurate, further studies will be necessary to verify that these techniques also work in cases with combined and complex severe 3D osseous deformations with variable portions of torsional (rotational), mediolateral (varus/valgus), craniocaudal (ante-/pro- and re-curvature) (17), and translational components. The last two were not addressed with this technique yet. Precise determination of three-dimensional limb alignment is especially interesting for patients with severe three-dimensional limb deformities (17), and therefore, proof of accuracy and precision in severely three-dimensionally deformed bones is a prerequisite prior to the establishment of any reference values.

## 5. Conclusion

In conclusion, by application of a three-dimensional CT-based technique using a 3D coordinate system, accuracy to measure normal and abnormal canine femoral torsion angles could be proved in a canine femoral torsional deformity model, and precision could be demonstrated for canine femoral inclination, torsion, and varus angle measurements in CT data of dogs that were scanned in a clinical setting.

## Data availability statement

The raw data supporting the conclusions of this article will be made available by the authors, without undue reservation.

## Ethics statement

Ethical review and approval was not required for the animal study because we used plastic bone models of canine femoral bone for the experimental part of the study. The clinical CT data were retrospectively selected and retrieved from the hospital image archive (PACS) from patients that were scanned unrelated to that study based on a clinical indication (presurgical evaluation and planning). All prior CT scans were performed within the normal clinical routine unrelated to this project on dog patients with a medial orthopedic indication for CT and with consent of the owners. Written informed consent for participation was not obtained from the owners.

## Author contributions

AB contributed to the conception and study design, development of methodology, measurements and data acquisition (operator 2, observer 2), analysis and interpretation, as well as draft, revisions, approval, and submission of the article. BS performed measurements and data acquisition (operator 1, observer 1), analysis and interpretation of data, and as well as the final approval of the article. MZ contributed to experimental, clinical CT scans, and as well as the final approval of the completed article. SR selected and calculated the statistical tests, revised the article for intellectual content, and approved the final article. AM-L contributed to supervision, clinical patient acquisition, orthopedic examinations and surgeries in the dogs with patellar luxation, revision of the article for intellectual content, and approval of the final article. All authors contributed to the article and approved the submitted version.
