# Supplementary material for: Computed tomographic angular measurements using a bone-centered three-dimensional coordinate system are accurate in a femoral torsional deformity model and precise in clinical canine patients
Source: Front Vet Sci. 2023 Apr 17;10:1019216. doi: 10.3389/fvets.2023.1019216 (PMC10149667; doi:10.3389/fvets.2023.1019216)
Supplement: Supplementary file 1 [file Table_1.DOCX]

**Supplementary Table S1.** Results of the comparison of independent goniometer measurements between two individual observers in 10 randomly preset torsion angles in a canine femoral torsional deformity model (interobserver agreement).

| Number of torsion and measurement | goniometer measurements of a randomly preset femoral neck torsion angle | |
| --- | --- | --- |
|  | observer 1 | observer 2 |
| 1 | 18° | 19° |
| 2 | 42° | 42° |
| 3 | 26° | 30° |
| 4 | 29° | 30° |
| 5 | 2° | 2° |
| 6 | 31° | 31° |
| 7 | 74° | 75° |
| 8 | 55° | 57° |
| 9 | 83° | 84° |
| 10 | 8° | 8° |
